# Slimming World in Stop Smoking Services (SWISSS): study protocol for a randomized controlled trial

**DOI:** 10.1186/1745-6215-14-182

**Published:** 2013-06-19

**Authors:** Deborah Lycett, Paul Aveyard, Andrew Farmer, Amanda Lewis, Marcus Munafò

**Affiliations:** 1 Department of Health Professions, Faculty of Health and Life Sciences RC131, Coventry University, Priory Street, Coventry CV1 5FB, UK; 2 Department of Primary Health Care Sciences, University of Oxford, Radcliffe Observatory Quarter, Woodstock Road, Oxford OX2 6GG, UK; 3 School of Experimental Psychology, University of Bristol, 12a Priory Road, Clifton, Bristol BS8 1TU, UK

**Keywords:** Weight gain, Smoking cessation, Prevention, Obesity, Commercial weight management provider

## Abstract

**Background:**

Quitting smokers gain weight. This deters some from trying to stop smoking and may explain the increased incidence of type 2 diabetes after cessation. Dieting when stopping smoking may be counterproductive. Hunger increases cravings for smoking and tackling two behaviours together may undermine quitting success. A meta-analysis of randomized controlled trials (RCTs) showed individualized dietary support may prevent weight gain, although there is insufficient evidence whether it undermines smoking cessation. Commercial weight management providers (CWMPs), such as Slimming World, provide individualized dietary support for National Health Service (NHS) patients; however, there is no evidence that they can prevent cessation-related weight gain.

Our objective is to determine whether attending Slimming World from quit date, through referral from NHS Stop Smoking Services, is more effective than usual care at preventing cessation-related weight gain.

**Methods:**

This RCT will examine the effectiveness of usual cessation support plus referral to Slimming World compared to usual cessation support alone. Healthy weight, overweight and obese adult smokers attending Stop Smoking Services will be included. The primary outcome is weight change in quitters 12 weeks post-randomization. Multivariable linear regression analysis will compare weight change between trial arms and adjust for known predictors of cessation-related weight gain.

We will recruit 320 participants, with 160 participants in each arm. An alpha error rate of 5% and 90% power will detect a 2 kg (SD = 2.5) difference in weight gain at 12 weeks, assuming 20% remain abstinent by then.

**Discussion:**

This trial will establish whether referral to the 12-week Slimming World programme plus usual care is an effective intervention to prevent cessation-related weight gain. If so, we will seek to establish whether weight control comes at the expense of a successful quit attempt in a further non-inferiority trial.

Positive results from both these trials would provide a potential solution to cessation-related weight gain, which could be rolled out across England within Stop Smoking Services to better meet the needs of 0.75 million smokers stopping with NHS support every year.

**Trial registration:**

Current Controlled Trials ISRCTN65705512

## Background

Weight gain is a well known consequence of smoking cessation [1,2]. It may deter smokers from attempting to quit [3] and offsets some advantages of giving up smoking. Smoking cessation-related weight gain partly explains the finding that the incidence of type 2 diabetes is increased by up to 73% in the years after cessation [4-6] and there is a 30% increased risk of hypertension [7] compared to continuing smoking. Quitters who gain weight have less improvement in lung function than those who avoid weight gain [8].

An ideal intervention to prevent weight gain would begin at the time of quitting, because weight gain is most rapid initially and then the rate of gain slows [9,10]. Qualitative findings suggest that, in the overweight population, at least, waiting until a quit attempt is established before addressing weight is unacceptable [11].

However, preventing weight gain early in cessation is controversial. The main reason quit attempts fail is due to quitters succumbing to their cravings to smoke. Evidence suggests that hunger increases urges to smoke [12,13] and people who gain most weight are more likely to succeed in quitting smoking [14]. This suggests that avoiding hunger and ameliorating cigarette cravings with food may enhance smoking abstinence and this has become standard advice on how to quit, with healthy food choices often advocated.

We investigated the effectiveness of different interventions to prevent weight gain during cessation in a recent Cochrane review update [15]. General dietary education to reduce energy intake through eating a low fat, healthy diet and increasing exercise did not prevent weight gain compared to standard smoking cessation behavioral support. Furthermore, there was a statistically significant reduction in abstinence at 12 months (Table 1). However, an individually tailored plan to reduce energy intake and increase exercise, with regular monitoring and adaptation of individual goals, reduced weight gain at 6 and 12 months without a statistically significant reduction in abstinence rates (Table 1), although the sample size is too small to be conclusive.

**Table 1:** Effects of dietary interventions on weight change and abstinence during smoking cessation

**Intervention compared to standard smoking cessation**	**Mean difference in weight change (kg (95% CI))**		**Abstinence (RR (95% CI))**	
	**End of treatment**	**At 12 months**	**End of treatment**	**At 12 months**
General lifestyle and calorie reducing dietary advice	−0.04 (−0.57, 0.50)	−0.21 (−2.28, 1.86)	0.90 (0.76, 1.06)	0.66 (0.48, 0.90)
Individually tailored dietary and lifestyle advice	−1.11 (−1.93, −0.29)	−2.58 (−5.11, −0.05)	1.11 (0.84, 1.46)	0.74 (0.39, 1.43)

CI, confidence interval; RR, risk ratio.

Commercial weight management providers (CWMPs) offer this type of individually tailored dietary and exercise plan. They have already been shown to be an effective way for people to achieve successful weight loss through referral from NHS primary care [16]. Therefore referral to a CWMP on prescription from the National Health Service (NHS) Stop Smoking Services may be an effective way to prevent smoking-related weight gain without a detrimental effect on quitting success. The first step to investigating this is to run a trial to assess whether this is an effective way to prevent weight gain during smoking cessation.

We will use the results of such a trial to inform a larger trial powered to investigate non-inferiority on smoking cessation rates. This will assess whether weight control comes at the expense of a successful quit attempt. This trial would require over 1,100 participants and is premature without first showing that the intervention can reduce weight gain, which is what we propose here. We will seek further funding for the larger trial to investigate effects on abstinence if the intervention reduces weight gain.

## Methods

### Design

This is an open label, phase II, randomized controlled trial (RCT) to compare standard stop smoking behavioral support with an intervention that will, in addition to providing standard stop smoking support, include personalized weight management support, provided by Slimming World.

This is an open label trial, so blinding patients and smoking cessation advisors to allocation to intervention or control is impossible. The primary outcome is objectively measured weight and therefore the scope for bias is limited. Stop smoking advisors, who will weigh participants, will not provide the weight control support and are unlikely to have a vested interest in interpreting weight change favorably. In addition, they will be required to follow a protocol that specifies outdoor coats and shoes only are removed before weighing.

### Setting

We propose recruiting participants from NHS commissioned Stop Smoking Services within Bristol and North Somerset in the first instance. Participating services located within pharmacies, general practices and community centers will display an A3 poster advertising the trial.

## Participants

### Inclusion criteria

• Daily smokers with expired CO >10 ppm.

• Aged 18 years or over.

• Body mass index (BMI) greater or equal to 23 kg/m^2^. We included participants with an ideal BMI, as well as those who are overweight in the trial. This is because we are not only seeking to help people who are overweight to manage their weight, but also to enable those who are a healthy weight to prevent the weight gain that is associated with smoking cessation. Without engaging in weight management strategies to counteract the physiological and behavioral responses to smoking cessation, 85% of people who quit smoking will gain weight, regardless of their starting BMI [17].

• Willing to be randomized to either the control or intervention arm, and willing and able to comply with the intervention and all study procedures.

• Able to understand and consent to study procedures, assessed at first appointment with the stop smoking advisor.

### Exclusion criteria

• Pregnant smokers.

• BMI <23 kg/m^2^. Mortality has been shown to be lowest in people with a 22 >BMI <25 [18], therefore preventing weight gain in those with lower BMIs may not lead to health gain.

• Any medical condition in which weight loss would be contraindicated, for example current course of chemotherapy.

• Currently losing weight, intentionally or unintentionally.

• Participating in any other intervention trial.

### Withdrawal criteria

#### Participant decision to withdraw from treatment and follow-up

Participants may choose to withdraw from treatment and/or follow-up at any time. Failure to attend treatment visits will not be considered as withdrawal from the whole trial, and these participants will still be contacted for follow-up appointments unless they have requested otherwise. We expect that participants who actively request to withdraw from the trial and no longer be contacted will be fewer than 5% of participants, and as such we will not replace these individuals.

#### Research team decision to withdraw

The intervention is based on the principles of healthy eating and we do not expect any adverse effects. Standard practice within the intervention is to monitor weight and provide feedback, and therefore an unhealthy rate of weight loss is unlikely to occur for long without correction. In the event that a participant develops a condition in which dietary energy restriction is ill-advised, for example embarking on a course of chemotherapy, they will be withdrawn from treatment. Data up to the time of withdrawal from treatment will be used unless the participant requests otherwise. Before beginning the trial, participants will have to consent to notifying the research team of such a change in medical history which is detailed on the consent form. We will ask the participant’s general practitioner to notify the research team of such changes in medical history.

### Randomization

A randomization sequence will be generated using computer software. Stratified randomization by stop smoking advisor with blocking within each stratum will be used to ensure balance. The blocks will be randomly ordered blocks of 2, 4 and 6. Participants will be randomized 1:1 to usual care or Slimming World with usual care after they have consented to take part in the study. Stop smoking advisors, who are unaware of the randomization sequence, will open sealed, numbered, opaque envelopes in turn, after consent and initial procedures to determine allocation. Online or telephone randomization is not practical in many smoking cessation clinics because they do not have access to computers and operate outside of normal office hours.

## Interventions and comparisons

### Trial treatment providers

Trained NHS stop smoking advisors will provide participants with standard smoking cessation support, which is intended to be identical in both arms of the trial. Weight control support will be provided by trained weight management counselors employed by Slimming World. Slimming World is a CWMP which follows the National Institute for Health and Care Excellence (NICE) criteria for clinical practice. It is commissioned by the NHS to provide a weight loss service to patients.

### Provision of standard NHS stop smoking support

All participants will receive standard NHS stop smoking support. This will be the withdrawal oriented behavioral support provided by NHS Stop Smoking Services, which increases the chance of a successful quit attempt by four-fold [19]. This consists of weekly behavioral support typically for 2 weeks before and until 4 weeks after quit day, focusing on key behavioral change techniques, and nicotine replacement, varenicline or bupropion are prescribed to relieve withdrawal symptoms.

**Figure 1:**
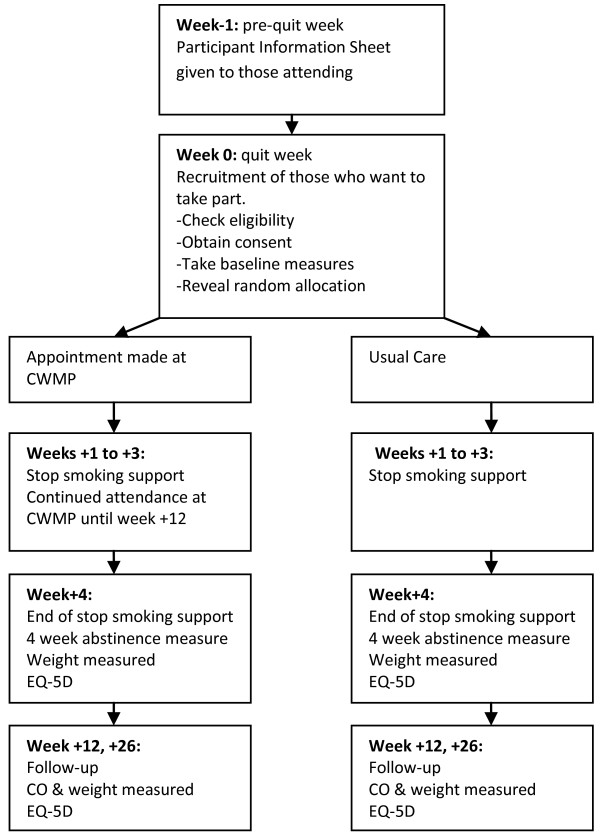
**Flow diagram showing flow of participants through trial and main procedures.** −, weeks before quitting; 0, quit week; +, weeks after quitting.

Standard stop smoking treatment consists of six sessions [20]. These are pre-booked when participants contact the Stop Smoking Service. Our trial will be nested within this usual practice as described below and shown in Figure 1.

### Trial procedures

#### Session 1: pre-quit session (week −1 prior to quit day)

During the first visit, as is usual practice, all smokers will set a quit date for 1 to 2 weeks in advance and their supply of medication to help them quit will be discussed. Smokers will be given the participant information sheet explaining the trial, providing potential participants enough time for consideration between this and the next visit. The information sheet states the opportunity to take part is entirely optional and that choosing not to participate will have no bearing on receiving usual stop smoking support.

#### Session 2: quit week (week 0)

At the second visit, smokers are usually ready to quit the next day and they will be either already taking varenicline or bupropion, or provided with nicotine replacement therapy at this visit.

Smokers will be asked whether they would like to participate in the trial. Before doing so, the stop smoking advisor will go through the trial information to make sure they have understood what is involved; for example they have to agree to attend Slimming World if randomized to it and be prepared not to go to a CWMP of their own volition if they are not randomized to it.

Those who decline to take part will be invited to give a reason. Those who agree to take part in the trial will provide written evidence of consent and will then be screened for eligibility by the stop smoking advisor. Baseline measures will be taken and they will be randomized into either usual care or Slimming World with usual care. The stop smoking advisor will provide those who are randomized to the intervention with a referral to Slimming World. This is to be taken and used at the local Slimming World group to coincide as near as possible with their quit date.

Baseline measures include a questionnaire asking for each participant’s age, gender, socioeconomic status, ethnicity, religion, daily cigarette consumption, and Fagerström test for nicotine dependence (FTND) score as usually measured by Stop Smoking Services. They will also be asked to complete the EQ-5D (EuroQol, Rotterdam, Netherlands) measure of health status and report whether they suffer from type 2 diabetes mellitus.

#### Sessions 3 to 5: weeks 1 to 3 post-quit (week +1, +2, +3)

The next three sessions will provide behavioral support for quitting in both the intervention and usual care group. The intervention group will also attend Slimming World.

#### Session 6: week post-quit (week +4)

This is typically the last visit to the Stop Smoking Service and when 4-week abstinence will be assessed. Weight will be measured and the EQ-5D will be completed by all participants at this visit.

#### Session 7: 12-week follow-up (week +12)

This corresponds with the end of weight management treatment. Weight and smoking abstinence will be measured. This session is important for determining the sample size needed for the non-inferiority trial which, as is standard in stop smoking trials [21], will require a 6-month follow-up to assess long-term smoking abstinence.

#### Session 8: 26-week follow-up (+26)

If data from 12-week follow-up shows a difference in mean weight, we will include a 26-week follow-up. The numbers of individuals who will return at this point will be recorded, and weight and smoking abstinence will be measured.

### Intervention

CWMPs are available ‘on prescription’ through general practices and provide 12 weeks of free membership of the group-based weight management class. There are three main providers offering services to the NHS: Rosemary Conley, Slimming World and Weight Watchers; the cost of a 12-week course to the NHS is about £60. Each of these providers are potentially suitable and their interventions have been described elsewhere [16]. In brief, each provides goal setting for weight, regular monitoring, and a tailored energy restriction using a either a food points system (Weight Watchers), food portion measures (Rosemary Conley), or controlled amounts of high energy foods and personal eating plans (Slimming World).

For this trial, we are using Slimming World, since this provider has been successful at improving health and reducing health risks associated with specific medical conditions through referral schemes within particular patient populations, some of which are designed to prevent excessive weight gain rather than focus on weight loss, for example during pregnancy [22,23]. Such an approach is particularly pertinent to quitting smokers who need to prevent cessation-related weight gain.

In addition to usual care, participants will be given a referral form for Slimming World when they attend their pre-quit visit. The stop smoking advisor will discuss times and locations of local group meetings, and attendance at a convenient group will be agreed. The participant will attend weight management sessions from their quit day (or as near to that date as possible). They will attend Slimming World for 12 weeks receiving support to lose weight or prevent weight gain. The choice of modest weight loss or weight gain prevention will depend upon whether a participant wants to lose weight or not, and whether or not the participant is overweight or obese at baseline.

Typically, Slimming World members lose 0.3 kg/week (or 0.4 kg/week in people who complete 10 of the 12 weeks) [24]. We know that on average 0.2 kg/week is gained with smoking during the first 12 weeks of quitting [10]. Factoring in this expected change in weight resulting from quitting, we would expect a mean weight loss of 0.2 kg/week. For participants who are overweight, a theoretical minimum weight loss goal may therefore be 2.4 kg or 3/4 stone over 12 weeks, although trials to date to prevent smoking cessation weight gain have not achieved as much as this (Table 1). Slimming World consultants typically agree weight loss targets with their members at the first appointment, so appropriate targets will be negotiated on an individual level within realistic margins. Slimming World consultants then work with their members to achieve this.

For participants whose starting BMI is 23 kg/m^2^, for example a 60 kg woman with a height of 1.62 m, the weight loss of 2.4 kg described above would result in a BMI of no less than 22 kg/m^2^, which is in the optimum range for longevity; this is why we have chosen a BMI less than 23 kg/m^2^ for our exclusion criteria.

Most people attending Slimming World do so with weight loss as their goal. For participants in this study who are only seeking to prevent further weight gain, they will be reassured that weight maintenance (+/−0.5 kg over 12 weeks (see section on sample size)) is an equally worthwhile target during smoking cessation and the participant information sheets for the trial will clearly explain this.

### Control

Participants are encouraged to quit smoking first, before tackling weight. Stop smoking advisors can advise satiating hunger with healthy foods, but they will not provide detailed or personalized advice on weight and will not set weight targets.

## Outcome measures

### Primary outcome measure

Change in weight from baseline to 12 weeks post-quit day in abstinent smokers. Abstinence is prolonged abstinence, defined according to the Russell Standard as self-report of no more than five cigarettes since 2 weeks following quit day and expired CO less than 10 ppm [21]. Portable validated scales will be provided to participating Stop Smoking Services. Training in measurement procedures and the trial protocol will be provided for all advisors. The same scales will be used at each clinic at all time points. All measures will be recorded on paper case report forms (CRFs) and later entered into a trial database.

### Secondary outcome measures

• Weight at 4 weeks post-quit date.

• Weight at 6 months from baseline.

• Abstinence at 4, 12 and 26 weeks. Abstinence will be defined as not smoking more than five cigarettes since 2 weeks after the quit day verified by expired CO <10 ppm at these visits (Russell Standard criteria). Abstinence will be recorded on the CRF and in the trials database.

• Participant acceptability.

#### Participant acceptability

Participant acceptability will be measured quantitatively by response, attrition rates and a questionnaire as described below:

• Rates of response to verbal invitation to take part by stop smoking advisors, including reasons for declining to participate.

• Rates of attendance at the commercial weight management meetings by participants randomized to this intervention.

• Rates of drop out from Stop Smoking Services in intervention compared to usual care.

• Rates of attendance at Stop Smoking Services for follow-up at 12 and 26 weeks.

• The above measures of acceptability according to starting BMI; for example are participants who are obese or overweight more accepting of the intervention which refers to Slimming World than those of a healthy weight?

• A questionnaire will be given to participants who decline to take a part, which will ask them to provide their age, gender, ethnicity, weight status, and reason(s) for declining using a series of closed answer responses (for example: ‘I do not want to take part in research’, ‘I do not think the intervention will work’, ‘I do not want to lose weight’) and one open ‘other’ category.

• All participants who drop out of treatment or follow-up will be asked, if they are willing, the reason for discontinuing using a series of closed answer responses (for example: ‘it was too hard to quit smoking’, ‘it was too hard to lose weight’, ‘I did not like the allocation I received’) and one open ‘other’ category.

#### Feasibility

Feasibility of running the trial within the Stop Smoking Services will be assessed by questionnaire. Participating services will be asked to rate the ease of adhering to the research procedures and describing any difficulties encountered, such as problems with recording data.

### Exploratory outcomes measures

• Self-reported incidence of diabetes.

• Change in EQ-5D.

• Cigarette withdrawal symptom scores as measured by the mood and physical symptoms scale (MPSS) [25].

• Associations of change in religious engagement (adapted from CSI-MEMO [26]) and religious coping (religious coping index (RCI) [27]) at baseline, and their change over time with tobacco withdrawal, quitting success and weight maintenance outcomes.

A recent systematic review of observational studies shows less smoking is associated with religion. Also, despite a higher BMI, religious involvement is associated with better physical and psychological outcomes [28]. The use of religion as a means to cope with health problems is a potential mechanism behind this association [27].

Clinical trials comparing weight loss interventions with a religious element to those without have shown increased physical activity [29], improved dietary quality [30] and greater mean weight loss [31,32] in participants receiving the additional religious element. Survey and qualitative data suggest smokers [33] and those who overeat [34] are receptive to incorporating a spiritual element into treatment programmes.

Although this evidence suggests a beneficial association and an openness to addressing spiritual needs in those who need to lose weight and stop smoking, many of these studies have been carried out in the Bible Belt, USA, and their applicability to the UK population needs to be determined.

## Statistics and data analysis

### Sample size

A meta-analysis of studies shows that smokers who achieve 12 weeks of abstinence gain around 2.5 kg (SD = 2.5) [10]. A Cochrane review to prevent weight gain when people stop smoking [15] showed that at 4 and 52 weeks post-quit there was a difference of 1.1 kg and 2.6 kg, respectively, between usual care and individualized dietary intervention (Table 1). This means that individualized dietary interventions prevented most of the expected weight gain at 4 weeks post-quit and half of it at 1 year post-quit. A realistic estimate is therefore that we may be able to prevent 80% (2 kg) of the expected weight gain at 12 weeks post-quit, that is the intervention group might gain only 0.5 kg on average. This would require about 32 participants per arm, 64 participants in total, with an alpha error rate of 5% and 90% power. Most smokers who attempt abstinence will relapse to smoking, probably lose any weight gained [9,31] and will not be included in the primary outcome assessment. Considering that approximately only 20% will remain abstinent at 12 weeks, we will need to randomize 160 participants in each trial arm and 320 participants overall.

### Analysis plan

#### Primary analysis

Change in weight between intervention and the control arm of the study will be compared using a *t*-test and then adjusted for known predictors of weight gain after cessation, including stop-smoking medication use, using linear regression analysis. Weight will be analysed in long-term abstainers because only long-term abstainers gain more weight over time than the rest of the population.

#### Secondary analysis

Abstinence will be analysed on an intention-to-treat basis. This phase II trial is underpowered to detect the difference between abstinence rates in the control and intervention arm. We will calculate the proportion of participants abstinent in each trial arm. Analysis of abstinence rates will assume participants who do not report for follow-up have reverted to smoking, which is standard practice. Quantitative measures of acceptability will be presented as descriptive statistics.

#### Exploratory outcomes

The association of exploratory outcomes with weight control and quitting success will be examined using regression modeling and on an intention-to-treat basis. We will calculate cost of intervention, but will not carry out any specific health economic modeling in this phase II trial, since it would be premature to do so. We need first to establish whether this weight management can prevent weight gain, before assessing the combined effects of the weight gain prevented and the effects on cessation rates in a phase III trial.

### Loss to follow-up

In accordance with the Russell Standard, we will conduct an intention-to-treat analysis and assume participants lost to follow-up are smokers. We will attempt to contact participants who do not attend. Reminder phone calls will also be made before 12-week and 6-month follow-up appointments.

### Trial closure

The end of the trial will be the date of the last follow-up of the last participant. Six-month follow-up is contingent upon data at 12-week follow-up.

## Study procedures

### Training

Stop smoking advisors will be trained in the research procedures for this study, in line with Good Clinical Practice (GCP) principles. Training will include how to screen participants, explain randomization, obtain consent and document data. Stop smoking advisors will be given the opportunity to practice via role play and will be observed doing so. In addition, stop smoking advisors will be provided with a ‘script’ and a prompt sheet for data recording for consultations that are beyond usual care.

In the case of any uncertainty (regarding suitability for inclusion), advisors will be able to contact the research team for clarification.

### Data handling, quality assurance, record keeping and retention

Data management will be undertaken according with the standard operating procedures (SOPs) of the Primary Care Clinical Research and Trials Unit (PC-CRTU) at the University of Birmingham, Edgbaston, UK. The PC-CRTU is fully compliant with the Data Protection Act 1998 and the International Conference on Harmonisation Good Clinical Practice (ICH GCP). The trial will be registered with the Data Protection Act website at the University of Birmingham. Participant identifiable data will be shared only within the clinical team on a need-to-know basis to provide clinical care, and to ensure good and appropriate follow-up. Patient identifiable data may also be shared with approved auditors from the research ethics committee (REC), the general practitioner, and NHS Research and Development (R&D). Otherwise, confidentiality will be maintained and no one outside the clinical or trial team will have access to either the case report forms or the database. On completion of the trial, data will be transferred to Modern Records, a secure archiving facility at the University of Birmingham, where they will be held for a minimum of 10 years and then destroyed.

### Case report forms (CRFs)

Data will be collected on a paper CRF and later entered into an anonymized database for analysis at the University of Birmingham. The database will contain agreed data codes, for example for missing data.

### Risk assessment

Healthy dietary and lifestyle advice will be individually tailored to create a mild energy deficit and gentle increase in activity. This advice will be given by appropriately trained Slimming World consultants. It is usual practice and considered very safe. Participants will be given the contact details of the trial team to report and discuss any concerns.

### Monitoring at study site

The principal investigator will assess adherence to the trial protocol through observation in clinics, for example to check advisors are not coercing smokers to take part, check the implications of randomization are adequately explained and to check the correct randomization procedure is followed. Record keeping will also be monitored, for example recording of weights and exhaled CO readings with random checks of the CRFs. Exhaled CO readings and participant existence will be verified by comparison to the source documents, that is stop smoking advisors records. Any deviations will be recorded, discussed with the stop smoking advisor concerned and corrected either immediately or at following clinics. The trial is potentially subject to audit by the appropriate regulatory authorities and monitoring by the lead Comprehensive Clinical Research Network, and therefore participants will be asked to provide consent to allow their records to be viewed by these authorities.

### Ethics approval

The study co-ordination centre has obtained approval from the Cornwall and Plymouth Research Ethics Committee. The trial will be conducted in accordance with the recommendations for physicians involved in research on human subjects adopted by the 18th World Medical Assembly, Helsinki, 1964, and later revisions.

### Participant consent

The process for obtaining participant informed consent will be in accordance with GCP. The stop smoking advisor and the participant shall both sign and date the consent form before any trial procedures begin. A copy of the signed form will be kept by the participant and the original will be retained at the trial site. A second copy will be forwarded to the general practitioner to file in the participant’s medical notes. The participant’s decision to take part in the trial is entirely voluntary. It will be explained to potential participants that they can withdraw consent at any time without penalty or affecting the quality or quantity of their future medical care.

Participants will be informed of any relevant information that becomes available that affects their participation in the study. Revised consent forms will be used if applicable and amended forms will be submitted to the main REC for favourable opinion prior to use. Revised informed consent forms will be signed by the parties specified above.

### Confidentiality

Participants’ identification data will be required for the registration process. The study co-ordination centre will preserve the confidentiality of participants taking part in the trial and is registered under the Data Protection Act 1998. Consent will be sought from patients for members of the study team, regulatory authorities and the relevant health provider to access patient medical records.

### Indemnity

The University of Birmingham holds public liability (negligent harm) and clinical trial (non-negligent harm) insurance policies, which apply to this trial.

### Sponsor

The University of Birmingham will act as the sponsor for this trial.

### Funding

Pump priming funds from the UK Centre for Tobacco Control Studies (UKCTCS) are funding this trial and the National Institute for Health Research School for Primary Care Research (NIHR-SPCR) paid the fellowship of the chief investigator while at the University of Birmingham.

### Audits

The trial may be subject to inspection and audit by The University of Birmingham, under their remit as sponsor, the study co-ordination centre and other relevant bodies, for example National Research Ethics Service.

### Participant payments

Participants will receive payment for travel and inconvenience at the following visits:

• 12-week follow-up: £5 voucher.

• 6-month follow-up: £5 voucher.

• Payments to NHS Stop Smoking Service.

The NHS Stop Smoking Service will be reimbursed for their time as appropriate from service support funding, for example to obtain participant consent.

### Treatment costs

Excess treatment costs of a 12-week membership at Slimming World will be provided to the trial free of charge in the form of vouchers by Slimming World.

## Discussion

This trial will establish whether referral to the 12-week Slimming World programme plus usual care is an effective intervention to prevent cessation-related weight gain. If so, we will seek to establish whether weight control comes at the expense of a successful quit attempt in a further non-inferiority trial.

Positive results from both these trials would provide a potential solution to cessation-related weight gain, which could be rolled out across England within Stop Smoking Services to better meet the needs of 0.75 million smokers stopping with NHS support every year.

## Trial status

Participant recruitment began in October 2012.

## Abbreviations

BMI: Body mass index; CI: Confidence interval; CRF: Case report form; CWMP: Commercial weight management providers; FTND: Fagerström test for nicotine dependence; GCP: Good Clinical Practice; ICH GCP: International Conference on Harmonisation Good Clinical Practice; LES: Local enhanced services; MPSS: Mood and physical symptoms scale; NIHR: National Institute for Health Research; NHS: National Health Service; NICE: National Institute for Health and Care Excellence; PC-CRTU: Primary Care Clinical Research and Trials Unit; R&D: Research and Development; RCI: Religious coping index; RCT: Randomized controlled trial; REC: Research ethics committee; SOP: Standard operating procedure; SPCR: School for Primary Care Research; UKCTCS: UK Centre for Tobacco Control Studies.

## Competing interests

DL has received hospitability from manufacturers of smoking cessation products, Pfizer Ltd, Tadworth, UK. DL’s institution has received smoking cessation products for use in a clinical trial from Johnson & Johnson Ltd, New Brunswick, NJ, USA. DL has received Slimming World membership vouchers for use in this trial. DL has received expenses and consultancy fees from the NHS and Universities for teaching about cessation-related weight gain. DL has received grant funding from UKCTCS and the NIHR-SPCR for research relating to cessation-related weight gain. PA has undertaken consultancy and research for manufacturers of smoking cessation medications in the past 5 years. AF receives funding from NIHR Oxford Biomedical Research Centre and his institution has received funding in respect of consulting work undertaken with Pfizer Ltd, manufacturers of varenicline, a stop smoking drug. AL has received hospitality from Weight Watchers. In the last 5 years, MM has received grant funding from Pfizer Ltd and varenicline, for research purposes.

## Authors’ contributions

DL designed the study and drafted the manuscript. AP, AF, AL and MM contributed to study design and manuscript revision. All authors read and approved the final manuscript.
